# Components of an Anticancer Diet: Dietary Recommendations, Restrictions and Supplements of the Bill Henderson Protocol

**DOI:** 10.3390/nu3010001

**Published:** 2010-12-30

**Authors:** Cynthia Mannion, Stacey Page, Laurie Heilman Bell, Marja Verhoef

**Affiliations:** Faculty of Nursing, The University of Calgary, 2500 University Drive NW, Calgary, Alberta T2N 1N4, Canada; Email: sapage@ucalgary.ca (S.P.); laheilma@ucalgary.ca (L.H.B.); mverhoef@ucalgary.ca (M.V.)

**Keywords:** anticancer diet, cancer, complementary and alternative medicine, supplements

## Abstract

The use of complementary and alternative medicines including dietary supplements, herbals and special diets to prevent or treat disease continues to be popular. The following paper provides a description of an alternative dietary approach to the self-management and treatment of cancer, the Bill Henderson Protocol (BHP). This diet encourages daily intake of raw foods, a combination of cottage cheese and flaxseed oil and a number of supplements. Some foods and food groups are restricted (e.g., gluten, meat, dairy). Early background theory that contributed to the protocol’s development is presented as is a summary of relevant evidence concerning the anti-cancer fighting properties of the individual components. Supplement intake is considered in relation to daily recommended intakes. Challenges and risks to protocol adherence are discussed. As with many complementary and alternative interventions, clear evidence of this dietary protocol’s safety and efficacy is lacking. Consumers of this protocol may require guidance on the ability of this protocol to meet their individual nutritional needs.

## 1. Introduction

The Bill Henderson Protocol (BHP) is a diet that is proposed to treat cancer and was first described in 2004 [1] (p. xiv). The primary daily components are raw fruits, vegetables, gluten free whole grains, legumes and a cottage cheese/flaxseed oil mixture [1] (pp. 102–103). Similar to other popularized and prescriptive diets, the BHP recommends restriction of some foods (*i.e.*, gluten and all dairy products other than cottage cheese) and inclusion of supplements. The supplement list is extensive; products containing beta glucan, barley grass, a multivitamin and mineral, and a nutrient combination of green tea, l-lysine, l-proline and vitamin C [1] (pp. 126–127). The BHP presents a dietary regimen with little evidence supporting its composition. The scientific background supporting the BHP is from 1950 and uses then known biochemistry that led to the cottage cheese/flaxseed oil combination. Little evidence beyond testimonials exists at this time but public interest persists. The following assessment provides the first in-depth analysis of the BHP.

Classified under the rubric of complementary and alternative medicine (CAM), the BHP is promoted as an alternative intervention to conventional cancer treatments. Cancer patients may decline or discontinue conventional treatments due to a limited understanding of effectiveness or concern with treatment side effects. However they too may be unaware of the uncertainties involved if they choose an alternative therapy. CAM information has been regarded as “empowering” as it broadens treatment and self-care options [2]. As well, some studies report that CAM is more common among those cancer patients who suffer from fears, uncertainties and dissatisfaction with the medical system [3]. The BHP holds widespread interest for those who have rejected or experienced failure with conventional cancer treatments [4,5].

There is potential for harm in embracing nonconventional nutritional therapies for disease treatment. The BHP is one of many diets utilizing a nutritional approach to decrease cancer risk or to treat cancer. Other examples include the Gerson Diet, Hoxsey Herbal Therapy, the macrobiotic diet, Manner Metabolic Therapy and Kelley Metabolic Therapy [6]. Little evidence supports the use and effectiveness of these diets to treat or prevent cancer. Typical of such diets, one food is incorporated as a primary constituent and other foods are eliminated because they are viewed as “harmful”. This can result in nutrient deficiencies especially if whole food groups are eliminated. Often insufficient energy intake results and little attention is paid to recommended nutrient intake from substantiated sources such as the Dietary Reference Intakes, Institute of Medicine [7].

The BHP is easily accessible to the public via website and the published books “*Cure your Cancer*” (ebook edition 2000, paperback edition 2003) and “*Cancer-Free: Your Guide to Gentle Non‑toxic Healing*” (first edition 2004, second edition 2007) [1] (p. ii). A third edition of *Cancer-Free* was released both as an ebook and paperback in November 2008 [8]. Through these books and online sources, readers are given justifications for the dietary recommendations of the BHP. These will be outlined, and discussed by any available data from current research. 

In May of 2009, the authors collaborated in the development of a survey disseminated to members of Bill Henderson’s database. Respondents provided their opinions and experiences regarding Bill Henderson’s products (e.g., books, newsletters, website, forums, *etc.*) and his protocol as well as describing their experiences with conventional medicine, complementary and alternative medicine generally. Some of their comments have been included here. Further details of this study will be published elsewhere (manuscript currently under review) [9]. 

## 2. The Basis for the BHP

The dietary suggestions, restrictions and supplements of the BHP address the processes Bill Henderson believes lead to the development of a variety of cancers. They include (1) lack of oxygen to the cells, (2) a weak immune system, (3) excessive acidity, and (4) toxicity in the body as a result of accumulation of tobacco, alcohol, asbestos, and teeth that have undergone root canal teeth [1] (pp. 44–45, 127–128). 

### Borrowing from the Budwig Diet: An Historical Basis for Lack of Oxygen

The BHP incorporates principal components of the Budwig Diet which was developed in the early 1950s by German chemist Dr. Johanna Budwig (1908–2003). She was a licensed pharmacist and published researcher [10,11,12] with doctorates in chemistry and physics [13] (p. 4). Budwig was searching for a diet to treat a variety of degenerative and autoimmune diseases. In the early 1950’s, while a senior research scientist in the German Federal Health Office, she observed that many of the cancer treating drugs being evaluated contained sulphydryl groups [13] (p. 10). Budwig believed that sulphydryl compounds were important to cellular metabolism, and in particular cellular respiration [13] (pp. 97–98). She focused her research on the development of cancer as a consequence of an aberration of cellular respiration, that in the absence of sulphydryl groups and/or a fatty acid partner, a low oxygen environment would be produced that would encourage cancerous cells to proliferate [13] (pp. 97–98). Note that it was in 1953 the Nobel Prize was awarded to Hans Krebs for his discovery of the Citric Acid Cycle [14]. 

Budwig’s theory is based upon the work of Otto Warburg (1883–1970) [13] (pp. 11–13). Warburg was an earlier Nobel Prize Laureate (1931) for the discovery of the nature and action of the respiratory enzyme [15], the first of the so-called yellow enzymes, or flavoproteins [16]. Warburg’s scientific efforts produced a large body of work and publications in highly respected journals such as Science (1928, 1956) [17,18,19]. According to Budwig, Warburg theorized that cellular respiration, like many chemical reactions, was dependent upon substrate availability, specifically a sulphydryl group and an unknown saturated fatty acid, which he failed to identify [13] (pp. 11–13). 

Budwig, although supportive of Warburg’s work, believed he was looking for the wrong fatty acid. From 1949 to 1952, Budwig and colleague H.P. Kaufmann developed new paper chromatography techniques to identify and quantify fatty acids, the success of which she says initiated widespread research into blood lipids [20] (pp. 5–6) [21,22,23,24,25,26,27,28,29,30,31,32]. Budwig describes how in 1953 she applied these techniques to blood samples of healthy and sick individuals, documenting the differences in fatty acid profiles [13] (p. 101) [24], making her one of the first scientists to question the health implications of fat consumption [20] (pp. 6–7). Modifying the type of dietary fat became the foundation for the development of the Budwig Diet [20] (p. 23). The modification and reduction of dietary fats in the prevention and treatment of cardiovascular disease and cancer has featured prominently in the last 50 years of dietary research [33,34]. 

## 3. A Rationale for the Dietary Components: Restoring an Oxygen Rich Environment

Budwig believed that patients with cancer required highly unsaturated fatty acids (HUFAs now referred to as PUFA’s or polyunsaturated fatty acids), specifically linoleic acid (LA) and linolenic acid (LNA) to act as raw materials for cell membrane formation and to drive cellular respiration [13] (pp. 97–98). Diets lacking these fatty acids offered limited substrate for reactions and resulted in an oxygen poor environment, impeding cellular metabolism. Flaxseed oil, which contains 18–20% LA, 58–60% LNA and lesser amounts of saturated and monounsaturated fat [35], is a key component of Budwig’s diet. It was Budwig’s theory that when the highly unsaturated fatty acids of flaxseed oil interacted with sulphydryl groups from cottage cheese, the stored energy in the fatty acids would be released remedying the oxygen poor environment [13] (p. 102). Mechanisms of carcinogenesis have evolved since Budwig’s time. In the last twenty years, acceptance has grown for endogenous mechanisms of carcinogenesis including oxygen free radicals (OFR). OFR’s arise from the cellular oxygen reduction reactions and are highly reactive. They attack cell components such as lipids, damage DNA damage and induce mutations [36,37].

### 3.1. Increasing the Solubility of Fats

The sulphydryl groups, cysteine and methionine, in cottage cheese, were described by Budwig as substances that facilitated the mobilization of fat by increasing solubility [20] (p. 30).Through her experiments using paper chromatography Budwig found that blending sulphydryl containing cottage cheese with flaxseed oil would improve the solubility of the flaxseed oil, a reaction that did not occur with the saturated fats derived from pork fat. She reasoned that the sulphydryl groups in the amino acids, hydrogen bonded with the unsaturated fatty acids, forming a lipoprotein [13] (p. 20). Lipoproteins are the building blocks of the phospholipid bilayer, or as Budwig called them “the external skin of the cell” [20] (p. 17). The proper function of cellular membranes is vital as it mediates the flow of materials in and out of the cell. Budwig felt the combination of these two substances (*i.e.*, cottage cheese and flaxseed oil) was important because the bond created by the opposing charges generated the “electromotor force” of a lipoprotein [13] (pp. 99–100), which she claimed provided the only path for fast and focused transport of electrons in biological systems [13] (p. 92). It was not until 1978 that Peter Mitchell received a Nobel Prize for his work on energy production in mitochondria [38].

### 3.2. Restrictions of the Budwig Diet

Processed fats, such as the hydrogenated fat found in margarines were prohibited in the Budwig diet [1] (pp. 104–106). Budwig believed these solid fats were oxygen inert [20] (pp. 9, 21). She also opposed the use of synthetic supplements, such as single vitamins or multivitamin compounds, claiming that synthetic nutrients did not function well in the body [13] (p. 43). Further, she felt that the use of synthetic antioxidants would block the restoring effect she believed flaxseed oil had on cellular respiration [13] (p. 50). There is some recent evidence that the potency of antioxidant effects in plant cells may differ between natural and synthetic vitamin E, but Budwig could only have suspected this [39]. Promotion of synthetic supplements is the major difference between the BHP and the original Budwig diet. The BHP suggests many vitamin supplements, some exceeding tolerable upper intake levels.

## 4. Cancer and the Budwig Diet

A conventional cancer theory claims that cancer is a disease involving rapid or uncontrolled cellular division as a result of changes in genetic information of cells [40] (p. 31). Errors in replication can either kill a cell or cause mutations that may lead to cancer [40] (p. 31). Budwig felt that cancer was a result of incomplete cellular division. She claimed that cells would begin the process of mitosis, but fail to completely divide due to the lack of substrate required to make the lipoprotein rich cellular membrane. The product of incomplete division would then be quarantined by the body through the formation of a tumour [13] (pp. 13–14) [20] (pp. 17–20). This theory allegedly accounts for the polyploidy, or the presence of multiple sets of chromosomes that can be observed in cancer cells. Budwig further claimed that the biochemical activity of highly unsaturated fatty acids would accelerate the rate of cell division resulting in the self-destruction of formed tumours [20] (pp. 17–20) [13] (p. 97). Today, cancer is considered to be a complex multistep disorder, the result of a combination of factors including exposure to radiation and/or carcinogens (damage to DNA), infection, genetics, aging, immune function disorders, and lifestyle factors such as smoking [41] (pp. 998–1000).

## 5. Modifications of the Budwig Diet for Inclusion in the BHP

In *Cancer Free*, Bill Henderson recommends principal components of the Budwig Diet but adds a variety of other substances that would not have received Budwig’s endorsement. True to prescriptive diets that emphasize ritual [42], Henderson suggests precise amounts of the cottage cheese/flax seed combination (2/3 cup of 1% or 2% organic cottage cheese (no preservatives), 6 tablespoons flaxseed oil) to be eaten immediately to avoid oxidation [1] (p. 103). Stevia (a natural sweetener), almonds, strawberries or blueberries are suggested additions to improve palatability and provide antioxidants [1] (pp. 102–103). Contrary to Budwig’s recommendations, Henderson advocates the strict avoidance of alcohol and meat and encourages the use of artificial antioxidants [1] (pp. 57–59, 121) [13] (p. 50, pp. 117–118) [20] (p. 34). 

### Lipid Metabolism: A Modern Perspective

Since the 1950’s we have gained a greater understanding as to how all exogenous fats, regardless of type, are absorbed and transported. Through mechanical, enzymatic and hormonal factors dietary fats are melted, emulsified, lysed and absorbed. In the duodenum, the products of the partial lipid digestion, namely monoacyglycerols, lysolecithin, cholesterol and fatty acids, combine with bile salts to form micelles, that are small enough to access the intramicrovillus spaces of the intestinal membrane [43]. Bile salts render micelles sufficiently water soluble to penetrate the water layer bathing the enterocytes. Upon interaction with the brush border of these cells, the lipid contents of the micelle diffuse out of the micelle and into the enterocyte. Once in the enterocyte most of the products of fatty acid digestion are resynthesized to form triacylglycerols, phosphatidylcholine and cholesteryl esters. Short chain fatty acids enter portal circulation directly and are attached to albumin for transportation to other tissue sites [43]. Resynthesized lipids are joined with protein to form chylomicrons, apolipoprotein A and B [44] (p. 96). Chylomicrons are excreted from the enterocyte into the lacteals, which carry them to the thoracic duct and later into the bloodstream to the liver, muscle and adipose tissue [41] (p. 17). 

There was limited understanding of fat metabolism in 1950 to inform Budwig’s theory. While high density lipoproteins (HDL) and low density lipoproteins (LDL) had been identified, it wasn’t until the 1970’s that apopeptides were discovered and their role and the role of lipoproteins in general, was more clearly understood [45]. The discovery of the LDL receptor in 1974 (Brown and Goldstein) provided the missing link in the understanding of fatty acid transport and the potential role various fats could play in human health [46]. In the absence of this knowledge, Budwig erroneously concluded that sulfur containing amino acids methionine and cysteine were required for the solubility and transport of fatty acids. Sulfur atoms play a role in the formation of esters produced during fatty acid digestion and taurine from cysteine, is a component of bile salts [44] (pp. 95–101), but these were not the mechanisms upon which Budwig’s theory focused. 

Budwig was strongly opposed to the intake of processed fats. Today, we recognize these fats as *trans-*fats, most commonly produced during partial hydrogenation of vegetable oils to increase the shelf life of foods. *Trans*-fats have been associated with a number of adverse health effects [47,48]. The American Dietetic Association, the Institute of Medicine, US Dietary Guidelines, and the National Cholesterol Education Project recommend limiting dietary *trans*-fat intake from processed foods wherever possible based on evidence that has demonstrated that a 2% increase in energy intake from *trans*-fat is associated with a 23% increase in cardiovascular risk [49]. Health Canada has had a *Trans*-Fat Task Force since 2005 that is poised to mandate recommendations made in 2007 that the food industry limit the *trans-*fat content of vegetable oils and soft margarines to two per cent of the total fat content and five percent total fat content in other foods [50]. 

There is some evidence to support Budwig’s belief regarding different fats and health implications, which we know today to be an important consideration in the development of cardiovascular disease, specifically atherosclerosis [41] (pp. 878–880). But at the highest level of evidence, that being meta-analysis of randomized controlled trials, causality is less certain. In Hooper’s *et al.* (2001), systematic review of 27 randomized controlled trials (30,092 person years of observation) including modified or reduced fat intake, they reported a reduction of cardiovascular events of 16% (0.84; 0.72 to 0.99) and a reduction of cardiovascular mortality of 9% (0.91; 0.77 to 1.07) [34]. However, The Women’s Health Initiative Randomized Controlled Dietary Modification Trial involving almost 50,000 women over an 8 year period found no significant effect of a low-fat diet on risk of invasive breast cancer or total cancer incidence [51]. It would appear that other lifestyle factors including regular exercise, smoking cessation, weight reduction, low-fat diet, and reduced alcohol intake have the potential to decreased the risk of some cancers, but long term prospective studies are needed to establish causality. In 2005, Dietary Reference Intakes were published addressing dietary fat and other macronutrients [7]. The panel of authors state that there is insufficient evidence to set a defined level of fat intake at which risk of inadequacy or prevention of chronic disease occurs, but they recommend an estimate of total fat would be 20–35% of energy, and have termed that an Acceptable Macronutrient Distribution Range (AMDR) [7].

The use of flaxseed oil for the treatment of cancer has recently received scientific support but has not been studied as extensively as lignans in flax seed. Two studies published in 2010 have shown flaxseed oil to increase the effectiveness of two drugs used for the treatment of breast cancer, tamoxifen and trastuzumab, by increasing apoptosis and reducing cancer cell proliferation [52,53]. However, the use of flaxseed oil in conjunction with pharmaceutical drugs was never intended by Budwig or by BHP so these results cannot validate their claims. 

## 6. The Supplemental Components of the Bill Henderson Protocol

### 6.1. Beta-Glucan: Strengthening the Immune System

Beta-glucan is a complex polysaccharide found in the cell walls of bacteria and fungi [54]. Henderson suggests a baker’s yeast (*Saccharomyces cerevisiae*) derivative in a specified dose of 500 mg/50 pounds body weight taken in the morning in a fasted state [1] (pp. 126–127). This step, he purports will boost immune function, helping immune cells to recognize and destroy cancer cells [1] (p. 91). 

There is a growing body of research on the use of beta-glucan for immune stimulation and modulation, although the research conducted to date has primarily consisted of *in vitro* studies using human cancer cells lines and animal studies [55,56,57,58,59]. Application of the results to human cases is limited. A 2007 study reported a significant increase (from 48–50% to 61–69%) in monocyte production in 23 women with advanced breast cancer after 15 days of oral ingestion [60]. This has not been replicated to date. A recent review article by Chan, Chan and Sze (2009) reported that beta‑glucans of differing sizes and branching patterns may have variable immune potency and result in a variety of clinical effects. To date there are no high quality clinical trial data available on assessing the effectiveness of purified beta-glucans and cancer treatment [57]. 

### 6.2. Barley Grass: Countering Acidity

Barley grasses are the new sprouts of barley and contain a wide range of nutrients and plant hormones. Henderson encourages the use of barley grass, considered alkaline, to counter the excess acidity he believes to be a contributing factor in the development of cancer [1] (p. 119). In his book *Cancer-Free* (2007) Henderson makes a claim that barley grass contains 3000 enzymes that are used by the body [1] (p. 118). Enzymes are proteins and will be metabolized in the same way as other dietary proteins and denatured during digestion. The therapeutic use of supplemental enzymes to treat exocrine pancreatic insufficiency is supported, but the supplements manufactured for this purpose are pH sensitive and survive strong stomach acid to deliver concentrated, therapeutic doses [61], unlike barley grass derivatives. 

Specific evidence supporting the use of an alkaline diet or alkaline supplements in cancer is not available. Research on this issue as related to bone health and osteoporosis is somewhat contradictory. Arnett (2008) reported that diet or drugs that shift the body’s acid-base balance towards alkalinity may be useful for the treatment of bone loss disorders [62]. Research from Canada, also from 2008, showed a linear association between changes in calcium excretion in response to changes in acid excretion, but it could not be established that the calcium was coming from mobilized bone [63]. In a follow-up 2010 cohort study, it was concluded that low urine pH from acid excretion is not a predictor of osteoporosis risk [64]. How these results might translate to issues related to cancer has not been elucidated.

More specific to the BHP, a German study (2009) demonstrated that the use of a multimineral supplement containing alkaline ash minerals significantly increased the pH of subjects’ blood and urine [65]. If bodily pH does in fact play a role in cancer, this study does suggest that alkalizing supplementation could be useful, but this is preliminary research only. 

Although many studies on barley are available, there are no studies related specifically to barley grass. The nutritional composition of wheat grass is close to barley grass and in one pilot study of 60 participants, wheat grass reduced the haematological toxicity related to chemotherapy in breast cancer patients [66]. 

### 6.3. Nutrient Mixture: The Claim of Preventing Metastases

The BHP advocates the use of a nutrient mixture of vitamin C, the essential amino acid l-lysine and the non-essential amino acid L-proline. The therapeutic use of this combination was initially described by Dr. Mathias Rath and Dr. Linus Pauling in the 1990s [67,68] and is purported to counter the mechanisms that lead to the metastasis of cancer [69]. Green tea was added to the nutrient mixture as it was found to improve the effect [69,70]. In his book *Cancer-Free* (2007), Henderson makes the claim that this nutrient mixture will slow down or completely stop the spread of the cancer [1] (p. 114). 

The Dr. Rath Research Institute in California conducts research on patho-mechanisms including cancer, and on the beneficial effects of micronutrients in various chronic diseases. They have published over two dozen studies from 2004 to 2009 related to the use of the nutrient mixture for cancer [71,72,73,74,75,76,77,78,79,80,81,82,83,84,85,86,87,88,89,90,91,92,93,94,95,96]. These studies were performed on human cancer cell lines and mice models. Cancers studied include skin cancer, liposarcoma, glioma, osteosarcoma, testicular cancer, melanoma, lung, renal adenocarcinoma, cervical, mesothelioma, bladder, fibrosarcoma, ovarian, mammary, breast, pancreatic, prostate, colon, Ewing’s sarcoma and lymphoma [71,72,73,74,75,76,77,78,79,80,81,82,83,84,85,86,87,88,89,90,91,92,93,94,95,96]. The primary focus of these studies has been to assess the ability of this nutrient mixture to inhibit the expression of enzymes known as matrix metalloproteinases (MMPs). MMPs have been found to be up-regulated in nearly every type of human cancer and are correlated with advanced stage, invasive and metastatic cancers [97]. The studies conducted by the Rath Institute showed a dose dependent inhibition of MMP expression [71,72,73,74,75,76,77,78,79,80,81,82,83,84,85,86,87,88,89,90,91,92,93,94,95]. 

Conversely, the only independent study testing this nutrient mixture’s effect on cancer is a recent German study involving of an animal model of neuroblastoma. Results suggested that this nutrient mixture was ineffective when tested on mice with induced tumours and spontaneous liver metastases [98]. A greater amount of objective, independent review of this nutrient mixture would help provide a basis for more firm conclusions to be drawn. 

Studies involving independent components of the mixture do exist. Demeule, Brossard, Page, Gingras and Beliveau (2000) published a study confirming the use of green tea polyphenols to inhibit MMP activity in rat and human tissue, with catechins epigallocatechin gallate and epicatechin gallate showing the greatest level of MMP inhibition [70]. 

Henderson credits Dr. Rath’s work and suggests including the nutrient mixture, but does not recommend Rath’s brand of expensive supplements [1] (pp. 113–114) (Table 1). Rath’s supplement lacks external investigation, raising the possibility of bias for his findings. 

**Table 1:** Specific brands and doses for supplements suggested in the BHP [1] (pp. 126–127).

Component	Source recommended by BHP	Daily dose recommended while on the BHP
Cottage cheese	organic, low fat	2/3 Cup (mixed with flaxseed oil)
Flaxseed oil	Barleans	6 Tablespoons (mixed with cottage cheese)
Beta glucan	Transfer Point	500 mg/23 kg body weight
Barley grass	Green Supreme	20 tablets divided into three doses
Heart plus	Our Health Coop	2 capsules/TID, to be taken with green tea extract
Green tea extract	Our Health Coop	1 capsule/TID, to be taken with Heart plus
Daily advantage	Mountain Home Nutritionals	1 packet/BID (8 capsules/packet)

### 6.4. Multivitamin/Mineral Supplement

The inclusion of this supplement in the BHP is part of the megavitamin trend popularized in the seventies. The multivitamin/mineral supplement is a combination including 65 vitamins, minerals, essential fatty acids, amino acids, anti-oxidants, digestive enzymes, herbs and “superfoods” (Table 2 and Table 3). A comparison of this combination to the recommended Daily Recommended Intakes (DRI) for males and females ages of 51–70 years is provided [7]. Based on data collected from the 2009 survey of BHP consumers, the 51–70 years age range were the most frequent users of the BHP [9]. 

**Table 2:** Supplemental nutrient intake recommended in the Bill Henderson Protocol compared to Institute of Medicine’s DRIs.

Nutrient	Total daily amount advised in the BHP [1] (pp. 57–59)	Daily recommended intake (male 51–70 y) [7]	Daily recommended intake (female 51–70 y) [7]	Tolerable upper intake level (adults 19–70 y) [99]
Vitamin A	3030 µg/10000 IU *	900 µg or 2970 IU	700 µg or 2310 IU	3000 µg/day
Vitamin C	4000 mg *	90 mg	75 mg	2000 mg/day
Vitamin D	40 µg/1600 IU	10 µg or 400 IU	10 µg or 400 IU	50 µg/day
Vitamin K	120 µg **	120 µg	90 µg	ND
Vitamin E	800 IU/536 mg	15 mg	15 mg	1000 mg/day
Thiamin	100 mg **	1.2 mg	1.1 mg	ND
Riboflavin	100 mg **	1.3 mg	1.1 mg	ND
Niacin	352 mg *	16 mg	14 mg	35 mg/day
Vitamin B6	220 mg *	1.7 mg	1.5 mg	100 mg/day
Folic Acid	800 µg	400 µg	400 µg	1000 µg/day
Vitamin B12	200 µg **	2.4 µg	2.4 µg	ND
Biotin	600 µg **	30 µg	30 µg	ND
Pantothenic acid	300 mg **	5 mg	5 mg	ND
Calcium	2000 mg	1200 mg	1200 mg	2.5 g/day
Iodine	200 µg	150 µg	150 µg	1100 µg/day
Magnesium	1000 mg *	420 mg	320 mg	350 mg/day
Zinc	40 mg	11 mg	8 mg	40 mg
Selenium	400 µg	55 µg	55 µg	400 µg/day
Copper	4000 µg	900 µg	900 µg	10000 µg/day
Manganese	20 mg *	2.3 mg	1.8 mg	11 mg/day
Chromium	400 µg **	30 µg	20 µg	ND
Molybdenum	200 µg	45 µg	45 µg	2000 µg/day
Potassium	200 mg	4.7g	4.7g	ND
Choline	200 mg	550 mg	425 mg	3.5 g/day
Vanadium	0.300 mg	ND	ND	1.8 mg/day
Boron	2 mg	ND	ND	20 mg
Quercitin	100 mg	ND	ND	ND
N-acetyl cysteine	100 mg	ND	ND	ND
Trace mineral complex	50 mg	ND	ND	ND
PABA	60 mg	ND	ND	ND
Inositol	200 mg	ND	ND	ND
Silica	52 mg	ND	ND	ND
Rutin	20 mg	ND	ND	ND
Hesperidin	20 mg	ND	ND	ND
Beta Carotene	15000 IU	ND	ND	ND
Tocotrienols	40 mg	ND	ND	ND
Coenzyme Q10	20 mg	ND	ND	ND
Alpha lipoic acid	20 mg	ND	ND	ND
Lutein	12 mg	ND	ND	ND
Lycopene	6 mg	ND	ND	ND
EPA (eicosapentaenoic acid)	200 mg	ND	ND	ND
DHA (docosahexaenoic acid)	300 mg	ND	ND	ND
Omega 3 fatty acids	100 mg	1600 mg	1100 mg	ND
Gamma linolenic acid	100 mg	ND	ND	ND
Ox bile	100 mg	ND	ND	ND
Pancreatin	100 mg	ND	ND	ND
Lipase	20 mg	ND	ND	ND
Cellulase	20 mg	ND	ND	ND
Maltase	20 mg	ND	ND	ND
Protease	20 mg	ND	ND	ND
Amylase	20 mg	ND	ND	ND

ND: No data are available on a recommended or maximum intake; * Intake recommended exceeds the tolerable upper limit (UL); ** Nutrient exceeds DRI, no UL has been established and caution is advised.

Six recommended nutrient supplements (vitamin A, C, B6, niacin, magnesium and manganese) exceed the tolerable upper intake level (UL), the highest level of daily intake that is likely to pose no risk of adverse health effects in most people [99]. Intake levels of vitamin A and C of this magnitude are known to cause nausea, vomiting, headaches, and insomnia and diarrhea and abdominal bloating respectively [41] (pp. 83, 113). Vitamin K, B12 thiamin, riboflavin, pantothenic acid, biotin and chromium do not have a UL established, but caution is advised when exceeding recommended daily amounts (RDA). The BHP suggested intake of these nutrients exceeds the RDA in some cases by 100 times or greater. Combinations of these nutrients in excess may result in adverse effects but remain unknown.

The “Herbal Superfood Booster” (Table 3) contains a list of herbal components and the daily dose suggested in the BHP [1] (pp. 57–59). Evaluations from the Natural Standard online database and the American Pharmaceutical Association on the efficacy of each are provided, with the increasing health risk represented by a letter (A–D) or number (1–5), respectively. 

**Table 3:** Daily advantage superfood herbal booster.

Daily advantage herbal	Daily dose suggested in the BHP	Natural standard grade	American Pharmaceutical Association’s numerical value
Spirulina	1500 mg	C (various)	4
Turmeric	400 mg	C *	3
L-Taurine	400 mg	B (nutritional deficiency)	N/A
Siberian ginseng root and extract	460 mg	C *	2
Bee pollen	200 mg	C **	3
L-Carnitine	200 mg	A (nutritional deficiency)	3
Royal jelly	100 mg	N/A	4
Astragalus	100 mg	C ***	3
Ginger root	100 mg	C **	1
Gymnema sylvestre	100 mg	B (diabetes)	2
Green tea extract	100 mg	C *	3
Panax ginseng	80 mg	C	2
Gingko biloba	20 mg	C ***	1

N/A: Herbal has not been evaluated by source; * For cancer treatment/prevention; ** For the side effects of cancer therapy; *** For cancer treatment/prevention and the side effects of cancer therapy.

Natural Standard was founded by clinicians and researchers to provide high quality, evidence-based information about complementary and alternative therapies [100]. An international collaboration of more than 100 academic institutions, Natural Standard uses a comprehensive methodology and reproducible grading scales, to provide information that is evidence-based, consensus-based, and peer‑reviewed, tapping into the collective expertise of a multidisciplinary Editorial Board [101]. 

The letter grades assigned by Natural Standard are based on the quality and quantity of evidence for the use of foods, nutrients and herbs for the treatment of various medical conditions. The letter grades are defined as [102]:

A- strong scientific evidence;
B- good scientific evidence;
C- unclear or conflicting scientific evidence;
D- fair scientific evidence;
F- strong negative evidence.

The American Pharmaceutical Association (APA) is a national, professional society of pharmacists whose mission is to support pharmacists in their ability to educate and advise the public on the safety and efficacy of medicines [103] (p. xi). In their publication *Practical Guide to Natural Medicine*, the APA assessed dietary supplements of herbs and amino acids. Numerical values were assigned to herbs and nutrients based on the quality and quantity of available research. The following is how each numerical value was defined:

1. Years of use and extensive, high-quality studies indicate that this substance is very effective and safe when used in commonly reported dosages.
2. According to a number of well-designed studies and common use, this substance appears to be relatively effective and safe when used in commonly reported dosages.
3. Studies on the effectiveness and safety of this substance are conflicting or there are not enough studies to draw a conclusion.
4. Research indicates that this substance will not fulfill the claims made for it, but that it is also unlikely to cause any harm.
5. Studies indicate that there is a definite hazard in using this substance, even in recommended amounts [103] (pp. 12–13).

## 7. Dietary Restrictions of the Bill Henderson Protocol: Reducing Toxicity

The BHP prohibits meat, dairy products (with the exception of cottage cheese), gluten, sugar, processed food and alcohol. Links between cancer and diet have been investigated [33,104,105,106], but it is difficult to make general conclusions and by extension, recommendations regarding the restriction of entire food groups. The BHP does not take into account calculation of energy needs, which is an important consideration for prescribed diets [107].

### 7.1. Meat

Conclusions about cancer risk and the consumption of meat are difficult to make given the state of current evidence. In a case control study from Uruguay (2009), 846 cases and 846 controls were followed estimating meat consumption and lung cancer risk. Total meat consumption of red meat and processed meat are reported to be associated with an increased risk of cancer, while total intake of white meat, poultry and fish did not increase risk [108]. Intake of red meat also appears to be associated with an increased risk of breast cancer [109,110] although the variables controlled in studies vary widely such as menopausal status and cooking practices, making it difficult to draw definitive conclusions [110,111]. The intake of saturated fat, found in higher amounts in animal products, has been linked to breast cancer [33]. Colon cancer risk has also been correlated with intakes of red meat, pan fried meat and processed meat mutagens [112]. Fish and specifically the consumption of omega 3 fatty acids, has been associated with a lowered cancer risk [33,113], and more specifically, decreased mortality from prostate cancer [114]. 

### 7.2. Dairy

A link between cancer and dairy products has not been established, although there are hypotheses about the risks of milk and milk product consumption associated with an increase in growth hormone/insulin like growth factor-1 that is triggered by the ingestion of milk protein [115]. The recent publication from the European Prospective Investigation into Cancer and Nutrition reported an association between high dairy intake, high serum concentrations of IGF-1 and an increased risk for prostate cancer [33]. Conflicting evidence exists regarding the role of dairy in the development of colorectal cancer. It has been reported that high dairy intakes during childhood resulted in a near tripling in the odds of developing colorectal cancer in adulthood [116]. Conversely, two recent studies suggest that a higher intake of dairy products is associated with a lower risk of colon cancer [117,118]. High calcium intake has also been correlated with a lower risk of colorectal cancer [117]. Fortified milk and milk products are also a source of vitamin D, which has been associated with a reduced risk of cancer [119,120]. The BHP recommends a supplement containing 40 µg/1600 IU of vitamin D [1] (pp. 57–59), four times the current daily recommended intake of vitamin D.

### 7.3. Gluten

Gluten, found in a variety of grains including wheat, does contain several proteins that are known causes of respiratory and food allergies as well as contact hypersensitivity, in certain individuals [121], but this reaction is not associated with an increased risk of cancer. 

Celiac disease is described as a small intestine mucosal disorder that is gluten dependent and often characterized by nutrient malabsorption, diarrhea and weight loss [122]. A known complication of celiac disease is the development of bowel cancers [41] (p. 713). A 2009 article reported that if celiac disease was first diagnosed later in life, lymphoma was detected more often than in cases of early diagnosis, the possible culprit being duration of exposure to gluten [123]. The same study also postulated that those with celiac disease may actually be at a lower risk of gastric and colon cancers than previously thought due the celiac characteristic symptoms of impaired absorption and quick excretion of fat, fat soluble substances, hydrocarbons and other potentially carcinogenic substances [123]. In the general population however, an increased risk of cancer from gluten ingestion has not been established. 

### 7.4. Sugar and Processed Food

Foods high in sugar can replace nutrient dense foods, delivering more calories and fewer nutrients. Poor nutrition can lead to numerous health consequences including anemia, delayed immune response and prolonged wound healing. Foods with a high glycemic index have been linked to insulin resistance, which has been correlated with an increased risk for pancreatic cancer [124,125]. In a 2009 cohort study intended to assess this risk, those with the highest intake of fructose and glucose were found to be at higher risk for pancreatic cancer [124]. Positive associations have also been made between diets with a high glycemic load and both esophageal adenocarcinoma [126] and breast cancer [127,128,129]. 

High sugar intake is associated with weight gain and obesity. A 2008 study reported that “an estimated 33,966 new cancers (4% of all estimated cancers) in males and 50,535 (7% of all estimated cancers) in females diagnosed in 2007, or 6% of all cancers, may be potentially attributable to obesity [130]. Without the impact of rising obesity rates, incidence rates might have declined (instead of remaining stable) from 1988–1994 to 2001–2004 for uterus, breast and certain other cancers” [130]. The following, by the World Cancer Research Fund and The American Institute for Cancer Research, is a list of mechanisms by which obesity can increase cancer risk as reported in their publication “Food, Nutrition, Physical Activity and the Prevention of Cancer: A Global Perspective”:

- Obesity causes an elevation of insulin and leptin levels, which can promote the growth of cancer. Elevated leptin levels are associated with colorectal and prostate cancer.
- Insulin resistance is increased, causing the pancreas to compensate by increasing insulin production. Hyperinsulinaemia is correlated with an increased risk of cancers of the colon and endometrium, and possibly of the pancreas and kidney.
- Higher levels of body fat increases the level of estradiol in men and women and may also raise testosterone levels in women. Increased levels of these sex steroid hormones are strongly associated with endometrial cancer and postmenopausal breast cancer and may increase risks for colon and other cancers.
- Adipose tissue promotes inflammatory factors in the body. Obese individuals have higher concentrations of interleukin-6, C-reactive protein and tumour necrosis factor. Leptin, which again is raised in cases of obesity, also functions as an inflammatory cytokine. Chronic inflammation can increase ones risk of cancer [40] (p. 39).

There is also a potential link between sugar and cancer involving the effect of sugar on the immune system. It has been reported that the daily ingestion of 75–100 g of simple sugars, including glucose, sucrose, fructose and honey has been found to induce a fifty percent drop in the activity of white blood cells for two to five hours [131]. The immune system plays a central role in the elimination of altered and unhealthy cells, including cancer cells. 

### 7.5. Alcohol

Alcohol intake has been associated with an increased risk for certain cancers including breast, rectal and pancreatic cancers [132,133,134]. There is conflicting data on the effect of alcohol consumption and prostate cancer. While heavy drinking, 50 g or more per day, has been positively associated with prostate cancer [135], 36 g or more per day has been shown to decrease the risk of benign prostatic hyperplasia [136], a marker for prostate cancer risk. A 2009 study evaluated lifetime alcohol exposure and its link to cancer risk in men and concluded that moderate and high alcohol consumption increased risk for cancers of the esophagus, stomach, colon, liver, pancreas, lung and prostate. Cancer association was strongest for beer and to a lesser extent spirits [137]. Red wine, on the other hand, may offer some protective benefits against a number of degenerative diseases. In a study looking at the effect of alcohol on breast density, a strong intermediate marker for breast cancer risk, red wine showed a consistent inverse association, unlike other types of alcohol [138]. The protective mechanism associated with red wine is often attributed to red wine’s content of resveratrol [139]. A recent study suggested that the benefits of red wine include, but are not limited to resveratrol, but may also involve its red pigments [140]. Clearly, the type and amount of alcohol consumed is a factor in cancer risk and supports Henderson’s recommendation for restriction.

## 8. Discussion

This paper has summarized the available evidence on the anti-cancer properties of the components of the Bill Henderson Protocol. Prospective longitudinal studies with intermittent measures are not available for testing the efficacy of the BHP. Some data exists on some of the individual components suggested as part of the protocol. The protocol was created by Bill Henderson, motivated by his own interest in curing cancer [1] (p. xiii). To date, much of the evidence for the BHP has taken the form of user testimonials [141,142]. The vulnerability of seriously ill people may increase their susceptibility to attaching truth to testimonials. Henderson attempts to dissuade potential BHP followers from investing in conventional therapies by challenging the trust consumers typically have in the expertise of conventional practitioners and suggesting that they alone can manage their illness [1] (pp. 1–4). People may believe that the BHP is as effective as conventional treatment and may choose to forgo such interventions, possibly significantly affecting their outcomes. 

As a large component of his six component diet, Bill Henderson suggests cottage cheese and flaxseed oil recommended by Dr. Johanna Budwig in the 1950s. While it is known that the essential fatty acids found in flaxseed oil, as well as methionine and cysteine, are important in human health, with modern advancements to our understanding in areas of human physiology, lipid metabolism and lipoproteins, Budwig’s fervent belief in these combined substances as cure for cancer appears overly simplistic. Although Budwig lived until 2003, and was actively lecturing until the late 1990s, there does not appear to have been any attempts to test her theories using modern technology or different source materials, such as fish oil. 

An additional constraint to the interpretation and understanding of Budwig’s work is that it was originally published in German. While English translations are available, they are primarily copies of lectures, letters and transcribed interviews. The foundations of her theory seem at times to be “lost in translation” making it difficult to review and evaluate her theories.

The information available on the supplements that are a part of the BHP is limited. The use of a beta-glucan supplement as an immune stimulant does appear to be supported by scientific research, although there is still much to assess about the value of differing beta-glucan sources and concentrations [55,57,58,60]. Similarly, scientific data on the use of barley grass for cancer is limited. Barley grass, like wheat grass, is commonly viewed as a highly nutritive food, but the benefits may be greatest when the grass is pressed into a juice and consumed fresh. The processing of the grass into a powder for use in capsules offers the benefits of convenience and perhaps for some, easier palatability, but might reduce some of its nutritive value. 

The combination of lysine, proline, ascorbic acid and green tea extract has been studied by The Dr. Rath Research Institute and based on their published work, has shown this nutrient mixture to be a promising anti-cancer proliferation agent [71,72,73,74,75,76,77,78,79,80,81,82,83,84,85,86,87,88,89,90,91,92,93,94,95]. This has been at least partially supported by research into the individual use of green tea extracts or ascorbic acid in cancer treatment [70,143,144,145,146,147]. However, the one independent published study on the use of the nutrient mixture as a whole showed it to be ineffective against the cancer studied (neuroblastoma) [98]. A greater amount of objective, independent research would help provide a broader look at the issue and benefit those looking to draw more firm conclusions. 

Adherence to the BHP comes with several challenges; the first being the high number of pills to swallow on a daily basis. If one strictly adheres to all the components of the protocol, a minimum of 46 pills and 6 tablespoons of flaxseed oil are required each day. For cancer patients with digestive difficulties, including nausea and/or obstructions, this may not be possible. A common side effect of consuming large amounts of oil is nausea and this may impede adherence in taking the full amount of flaxseed oil. Those people intolerant of dairy products may find the ingestion of cottage cheese challenging. If one is able to consume all the required components of the protocol, the doses of certain nutrients including vitamin A, C, B6, niacin, magnesium and manganese exceed the tolerable upper levels of intake. Depending on the individual, this may or may not produce side effects. 

The possibility of conventional drugs interacting with the protocol supplements may also be of concern. For example, those on anticoagulants may be at risk of agonistic effects from an increased intake of supplements such as vitamin E, ginger, Ginkgo biloba, ginseng and omega 3 fatty acids, which can interfere with the expected therapeutic effects [103] (pp. 269–271, 295) [148]. 

The dietary guidelines of the BHP are restrictive, eliminating some of the most common foods in the Western diet. For many, this drastic dietary change may be one of the greatest challenges to protocol compliance and there is conflicting data about the value of eliminating the restricted foods. The nutritional status of cancer patients may already be compromised by virtue of their disease symptoms and further dietary restrictions may have negative effects such as insufficient energy intake. Supplementation in excess of recommended dietary intakes may trigger adverse effects that are unexpected. Evaluation of the potential benefits of the BHP in comparison to its potential negative effects in this population needs to be undertaken. Moreover, potential protocol adherents are encouraged to forgo conventional treatment, albeit for a limited period of time, to try the BHP and delay or attenuate treatment with known benefits.

## 9. Conclusion

The Bill Henderson protocol is a dietary approach for the treatment and management of cancer. Presently, the protocol as a whole has not been evaluated, although varying amounts of evidence exist for some of its components. While there is some historical suggestion of a scientific rationale for this protocol, its claims remain unproven. Certainly further study is needed. Future investigations should consider whether this protocol meets the nutritional requirements of consumers with cancer and if it has any impact on morbidity and mortality in this patient group. Information on dietary information needs to be made available to consumer groups so that they may make informed choices when undertaking such self-management regimens.
